# Effect of Dynamic Interaction between microRNA and Transcription Factor on Gene Expression

**DOI:** 10.1155/2016/2676282

**Published:** 2016-11-10

**Authors:** Qi Zhao, Hongsheng Liu, Chenggui Yao, Jianwei Shuai, Xiaoqiang Sun

**Affiliations:** ^1^Department of Physics, College of Physics Science and Technology, Xiamen University, Xiamen 361005, China; ^2^School of Mathematics, Liaoning University, Shenyang 110036, China; ^3^Research Center for Computer Simulating and Information Processing of Bio-Macromolecules of Liaoning Province, Shenyang 110036, China; ^4^School of life science, Liaoning University, Shenyang 110036, China; ^5^Department of Mathematics, Shaoxing University, Shaoxing 312000, China; ^6^Zhongshan School of Medicine, Sun Yat-Sen University, Guangzhou 510080, China; ^7^Guangdong Provincial Key Laboratory of Orthopedics and Traumatology, The First Affiliated Hospital of Sun Yat-Sen University, Guangzhou 510000, China; ^8^School of Mathematical and Computational Science, Sun Yat-Sen University, Guangzhou 510275, China

## Abstract

MicroRNAs (miRNAs) are endogenous noncoding RNAs which participate in diverse biological processes in animals and plants. They are known to join together with transcription factors and downstream gene, forming a complex and highly interconnected regulatory network. To recognize a few overrepresented motifs which are expected to perform important elementary regulatory functions, we constructed a computational model of miRNA-mediated feedforward loops (FFLs) in which a transcription factor (TF) regulates miRNA and targets gene. Based on the different dynamic interactions between miRNA and TF on gene expression, four possible structural topologies of FFLs with two gate functions (AND gate and OR gate) are introduced. We studied the dynamic behaviors of these different motifs. Furthermore, the relationship between the response time and maximal activation velocity of miRNA was investigated. We found that the curve of response time shows nonmonotonic behavior in Co1 loop with OR gate. This may help us to infer the mechanism of miRNA binding to the promoter region. At last we investigated the influence of important parameters on the dynamic response of system. We identified that the stationary levels of target gene in all loops were insensitive to the initial value of miRNA.

## 1. Introduction

MicroRNAs (miRNAs) [1, 2] are a class of endogenous small noncoding RNAs that bind to partially complementary sequences in target mRNAs, negatively regulating their protein production in higher eukaryotes, plants, and animals [1, 3–5]. Many experimental studies have revealed that miRNAs can regulate various biological functions [6, 7], for instance, development and metabolisms [8]. Also, they have been demonstrated to be involved in many cellular signaling regulation processes, including apoptosis, proliferation, and differentiation [9–11]. Moreover, a lot of biological and clinical experiments have shown that miRNAs are involved in the initiation and development of many diseases [12, 13], such as cancers [14] and HIV [15]. More and more attention has been focused on the molecular mechanisms related to miRNAs and their functions [16].

The production of miRNA is regulated by certain transcription factors (TFs) that are also key regulators in gene expression. It has been demonstrated that miRNAs and TFs are often highly interacted in a dependent or independent manner [17]. Therefore, miRNA functions can be understood more clearly only in the context of regulatory interactions between TF and miRNA. Experimental data have demonstrated that gene regulatory networks are often constituted of some basic subcircuits involving feedforward or feedback loops [18], which are often called motif [19]. Feedforward loops (FFLs) have been shown to be a major member of biological network motifs. Many theoretical works [20–22] and experimental studies [23] have been conducted to investigate their structure and functions within the context of gene expression regulation. These studies focused on FFLs at the transcriptional level, in which gene expression is controlled by two regulatory TFs. Moreover, certain miRNA-containing motifs are often embedded in a lot of gene regulatory networks (GRNs). It has been known that all miRNAs operate through a repressive action on target mRNA. However, considering the interaction between miRNA and TFs, the role of miRNA in gene regulatory network is not simply repressive. Therefore, the investigation of the effect of interaction between TF and miRNA on gene expression is very important to help us understand the role of miRNAs in the GRN and disease.

Mathematical model is a powerful tool used to describe the biological systems and discriminate between different tentative mechanisms [24–36]. Several studies have examined the mechanisms of miRNA-containing motifs using mathematical models. Osella et al. [37] used a detailed analytical model and simulations to investigate the function of the miRNA-mediated FFL. Their analysis demonstrated that the incoherent version of such FFL motif can provide precision and stability to the overall gene expression program with an efficient noise control, given the existence of fluctuations in upstream regulators. Morozova et al. [38] developed a mathematical model containing nine known mechanisms of miRNA action and discriminated among different possible individual mechanisms based on the kinetic signatures. Duk et al. [39] analyzed three mathematical models, in which miRNA either represses translation of its target or promotes target mRNA degradation or is not reused but degrades along with target mRNA. They showed that different mechanisms of miRNA action lead to a variety of types of dynamical behavior of feedforward loops. However, none of previous studies examined the effects of dependence (AND gate) or independence (OR gate) between miRNA and TFs on gene expression.

In this paper, we developed a mathematical model to quantitatively analyze the dynamics of miRNA-containing FFLs and investigate the interaction between miRNA and TF on gene expression. We examined four FFLs, in which each contains AND gate or OR gate. We analyzed the different dynamical behaviors between AND gate and OR gate for each of these four FFLs. Our results showed that different mechanisms with respect to AND or OR gate might produce distinct dynamics of the GRN. In addition, we examined the relationship between response time of gene expression and certain parameters in the model. Finally we investigated the influence of important parameters on the response of system. Our study advances our quantitative understanding on the dynamic interaction between TF and miRNA, particularly, with AND or OR gate in the GRN, and provides some implications on the miRNA-mediated dieses.

## 2. Results

### 2.1. Mathematical Model of FFLs

**Figure 1:**
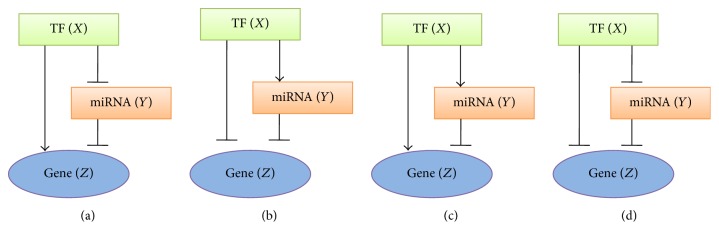
The coherent and incoherent feedforward loops. Arrows mean activation, the turned-over T-bars indicate repression. (a) Type 1 coherent FFL, TF activates target gene and represses miRNA synthesis. (b) Type 2 coherent FFL, TF represses target gene and activates miRNA synthesis. (c) Type 1 incoherent FFL, TF activates both target gene and miRNA synthesis. (d) Type 2 incoherent FFL, TF represses both target gene and miRNA synthesis.

Figure 1 illustrates the general structure of FFLs in miRNA-mediated gene transcription network, similar to that in [24–27]. The upstream transcription factor (TF) regulates the target gene via two parallel pathways: directly and by interaction with miRNA, which also regulates the target gene. Therefore, regulatory interactions in FFL create four possible structural topologies (Figure 1). Two of these configurations are named “coherent”: the sign of the direct regulation path from TF to gene is the same as the overall sign of the indirect regulation path from TF via miRNA to gene. The other two structures are termed “incoherent”: the sign of the direct regulation path is opposite to that of indirect path. We specify these configurations as type 1 or 2 coherent FFLs and type 1 or 2 incoherent FFLs, respectively. The biological network motif under investigation is described by 3 variables, the concentrations of transcription factor (*X*), miRNA (*Y*), and target gene (*Z*). The dynamical behavior of the FFLs is governed by the following equations:

(1) dXdt=k1−d1X,dYdt=v2fX,k12−d2Y,dZdt=v3gX,k13;Y,k23−d3Z.

The regulation function for an activator is *f*(*u*, *k*_*ij*_) = (*u*/*k*_*ij*_)^*n*^/(1 + (*u*/*k*_*ij*_)^*n*^) and for a repressor is *f*(*u*, *k*_*ij*_) = 1/(1 + (*u*/*k*_*ij*_)^*n*^), similar to that we used before in [40, 41]. *g*(*X*, *k*_13_; *Y*, *k*_23_) is the gate function, the mechanisms underlying miRNA-mediated repression are not clear so far, and for this reason we consider that the gate function has two forms. The gate function for an AND gate is *g*(*X*, *k*_13_; *Y*, *k*_23_) = *f*(*X*, *k*_13_)*∗f*(*Y*, *k*_23_), while for an OR gate we have *g*(*X*, *k*_13_; *Y*, *k*_23_) = *f*(*X*, *k*_13_) + *f*(*Y*, *k*_23_). For more details about the values of parameters and initial concentrations we use, see Tables 1 and 2.

### 2.2. Comparative Analysis of FFLs' Temporal Behavior under Different Gate Functions

We shall use for brevity the following abbreviations for the FFL identification: Co1 will mean type 1 coherent FFL, Co2 type 2 coherent FFL, In1 type 1 incoherent FFL, and In2 type 2 incoherent FFL, respectively.

**Figure 2:**
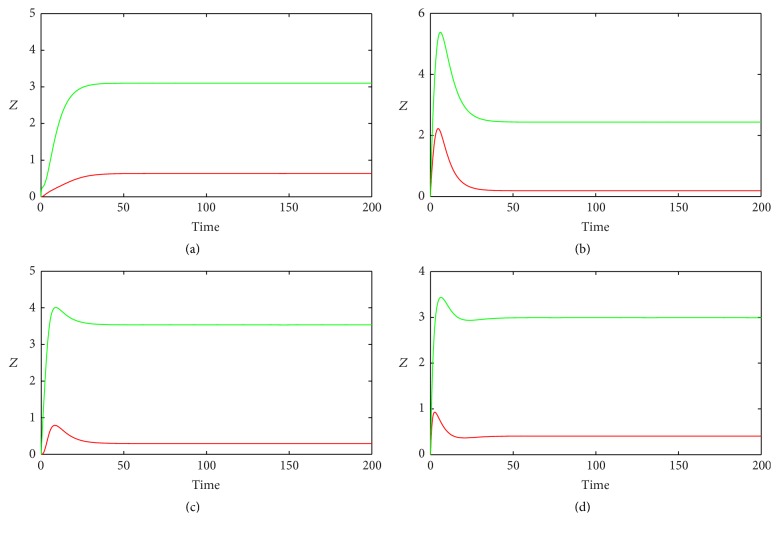
The time evolutions of *Z* in various FFLs with different gate functions when *k*_1_ is constant input. Types 1-2 coherent FFLs are shown in (a)-(b), while types 1-2 incoherent FFLs are given in (c)-(d). The red line corresponds to AND gate function, and the green line represents OR gate function. Here we fix *k*_1_ = 0.25.

Figure 2 shows the time courses of *Z* in various FFLs with different gate functions when *k*_1_ is constant number. Here *k*_1_ represents the basal synthesis rate of TF. The dynamics of target gene in Co1 loop has a form of increasing function and then tends to a constant vale (Figure 2(a)). The target gene profiles in Co2, In1, and In2 loops show pulse-like behavior due to repression mediated by miRNA (Figures 2(b), 2(c), and 2(d)). At the steady state, the concentrations of target gene in all the loops with AND gate are much lower than those with OR gate function. It is easy to understand this, because OR gate function makes the synthesis rate bigger than that of AND gate.

Living cells constantly have to respond to a changing environment. To understand how cells deal with a fluctuating environment, we need to know how cells transduce time varying signals. Next we consider the effect of providing the system with simultaneous pulse, a biological scenario which corresponds to continued exposure to environmental stimuli within a certain time range. Accordingly, we set *k*_1_ to be a piecewise constant function

(2) $$\begin{array}{c} k_{1}=\left\{\begin{array}{cc} 1 & 50\leq t\leq 100,\\ 0 & otherwise. \end{array}\right. \end{array}$$

**Figure 3:**
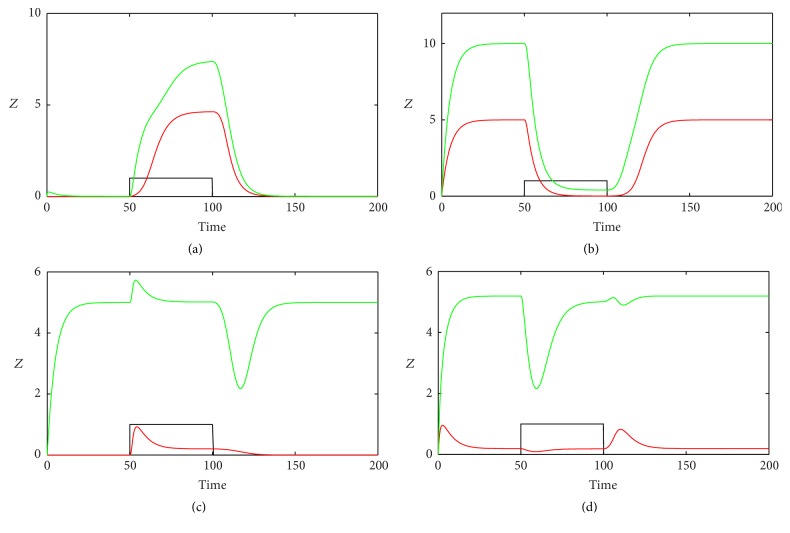
The time evolutions of *Z* in various FFLs with different gate functions in response to on and off steps of *k*_1_. Types 1-2 coherent FFLs are shown in (a)-(b), while types 1-2 incoherent FFLs are given in (c)-(d). The red line corresponds to AND gate function, and the green line represents OR gate function. *k*_1_ is set to 1 during the time between 50 and 100 and 0 in other time ranges (the black line).

Figure 3 shows the variations in the response of the output in the motifs. We first compare the kinetics of *Z* in Co1 and In1 loops (Figures 3(a) and 3(c)). When *k*_1_ turns on, we find out only the steady states of *Z* in Co1 and In1 loops with both gate functions rising up due to the direct activation of *Z* by TF (Figures 3(a) and 3(c)). But in In1 loop, *Z* first rises slightly and then falls down because TF inhibits *Z* by promoting miRNA. When *k*_1_ turns off, both the concentrations of *Z* in Co1 and In1 loops decrease, but *Z* in In1 loop with OR gate eventually grows again to the stationary level. We then compare the kinetics of *Z* in Co2 and In2 loops (Figures 3(b) and 3(d)); we observe that the concentration of *Z* in Co2 loop decreases as *k*_1_ turns on and increases as *k*_1_ turns off (Figure 3(b)). But *Z* in In2 loop with OR gate rises up again to the steady state level after *Z* falls down, as *k*_1_ turns on (Figure 3(d)), while *Z* in In2 loop with AND gate just slightly decreases when *k*_1_ changes to 1. *Z* in In2 loop with two types of gate functions shows pulse-like behavior after *k*_1_ turns to 0; however, the amplitude of *Z* in In2 loop with OR gate is much smaller than that with AND gate. From the subfigures in Figure 3, we can find that *Z* in In2 loop with AND gate is more robust in the presence of *k*_1_ addition, and *Z* in In1 loop with AND gate is more stable after an off step of *k*_1_.

**Figure 4:**
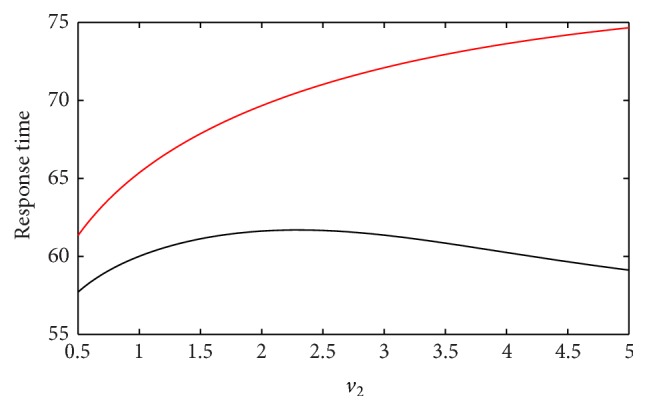
The response time is plotted against the variation of *v*_2_ in Co1 loop with different gate regulations. The red line corresponds to AND gate function, and the black line represents OR gate function. *k*_1_ is set to 1 during the time between 50 and 100 and 0 in other time ranges.

The response time is a measure of the time which a gene product takes to reach its physiologically determined steady state level. The speed of the response is characterized by the response time, which *Z* takes to reach half of its steady state level. Here *v*_2_ is the maximal activation velocity of miRNA by TF. In Figure 4, we study the relationship between the response time and *v*_2_ in Co1 loop with both gate regulations when providing the system with simultaneous pulse. We can observe that the response time has a form of increasing function as *v*_2_ turns bigger in Co1 loop with AND gate, which means the system responses more slowly as *v*_2_ increases. This is easy to understand; larger *v*_2_ induces more miRNA generation which further represses target gene synthesis, so the response time turns slowly. But for the case in Co1 loop with OR gate, the response time shows nonmonotonic behavior, which first climbs and then damps as further increasing *v*_2_. This indicates that there exists a value of *v*_2_ such that the system responses most slowly. To understand this, we need to refer to OR gate function we use. It is a nonmonotonic function as *v*_2_ increases, so the form of function decides the speed of the response of the system. Our result here might be useful to infer the mechanism of miRNA binding to the promoter region, whether or not the TF and miRNA compete for binding to the target gene. Also, we obtain that the response of gene expression in Co1 loop with OR gate is faster than that in Co1 loop with AND gate during the period of *v*_2_ changing.

### 2.3. Variations of Parameters on the Response of System

It is known that the model coefficients might affect the dynamical behavior of FFLs. Therefore, we further examine how the changes in parameters affect the temporal behavior of the target gene. We investigate the effect of changes in *v*_2_, *d*_2_, *k*_1_, and *d*_1_ on the dynamical behavior of *Z*.

**Figure 5:**
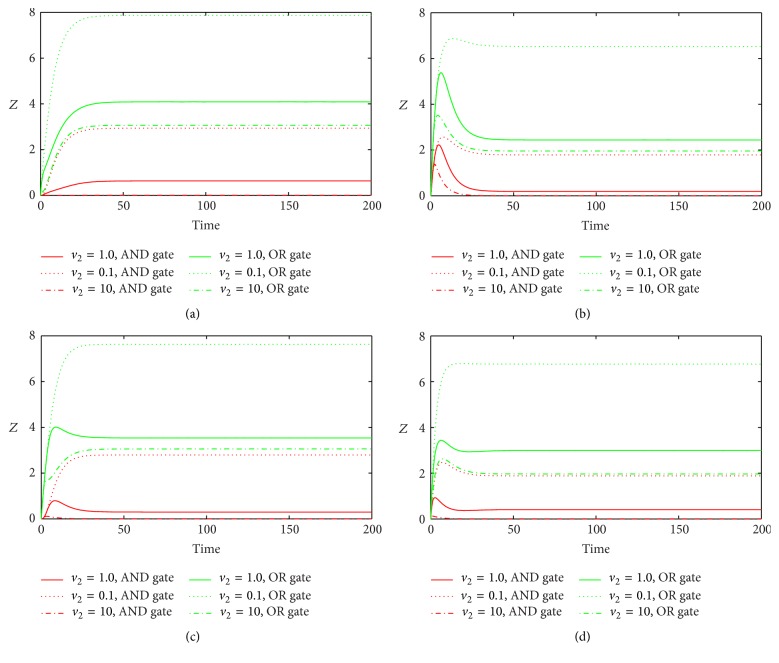
The time evolutions of *Z* in various FFLs with different gate functions in response to variation of *v*_2_. Types 1-2 coherent FFLs are shown in (a)-(b), while types 1-2 incoherent FFLs are given in (c)-(d). The red line corresponds to AND gate function, and the green line represents OR gate function. Here we fix *k*_1_ = 0.25.

**Figure 6:**
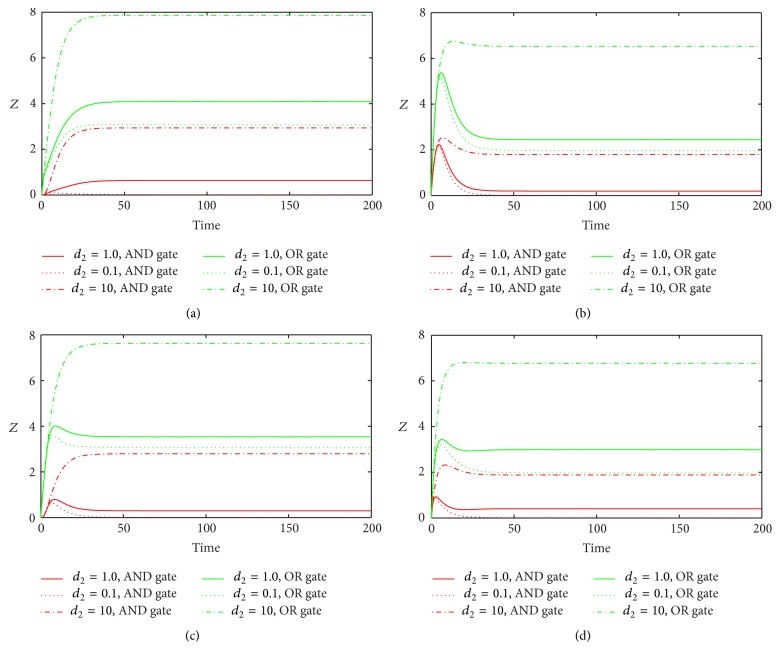
The time evolutions of *Z* in various FFLs with different gate functions in response to variation of *d*_2_. Types 1-2 coherent FFLs are shown in (a)-(b), while types 1-2 incoherent FFLs are given in (c)-(d). The red line corresponds to AND gate function, and the green line represents OR gate function. Here we fix *k*_1_ = 0.25.

Figure 5 shows the time course of *Z* in various FFLs with different gate functions in response to variation of *v*_2_. We choose three typical values of *v*_2_: the original value, 10-fold, and 0.1-fold of *v*_2_. We find that bigger *v*_2_ induces less expression of target gene when *Z* reaches the steady state. We can understand this from the interaction relationship in Figure 1. Larger *v*_2_ results in more miRNA generation which further represses target gene synthesis, so at last less target gene was observed. Parameter *d*_2_ is the degradation rate of miRNA. For the influence of *d*_2_, the situation is opposite, in which bigger *d*_2_ results in higher level of gene expression after it gets to the stationary level (Figure 6). This is because that larger *d*_2_ induces less miRNA generation, which results in less inhibition of miRNA on *Z* synthesis.

**Figure 7:**
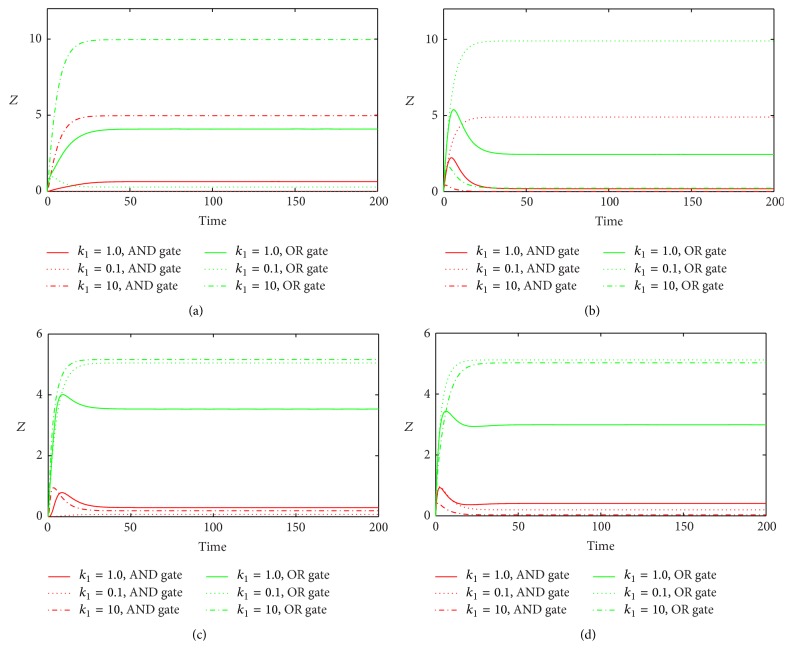
The time evolutions of *Z* in various FFLs with different gate functions in response to variation of *k*_1_. Types 1-2 coherent FFLs are shown in (a)-(b), while type 1-2 incoherent FFLs are given in (c)-(d). The red line corresponds to AND gate function, and the green line represents OR gate function. Here we fix *k*_1_ = 0.25.

**Figure 8:**
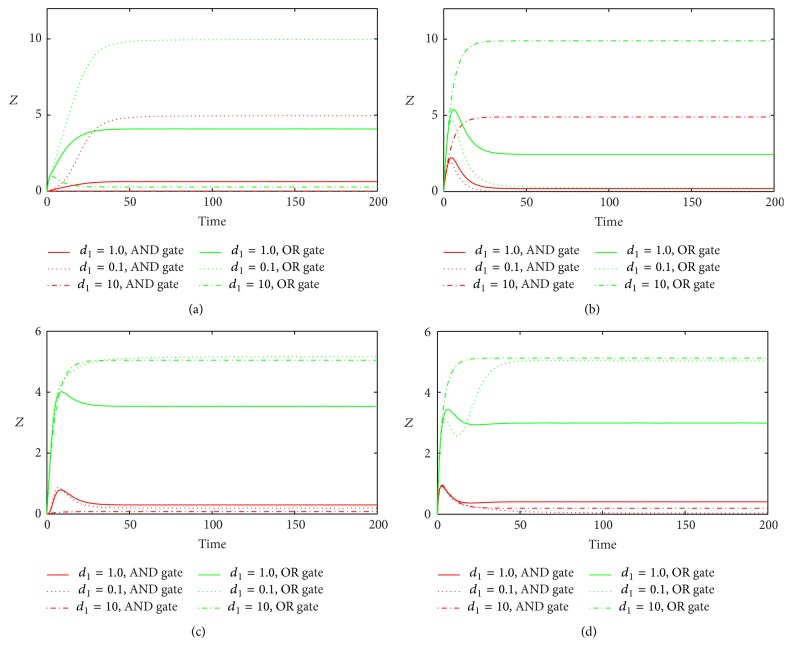
The time evolutions of *Z* in various FFLs with different gate functions in response to variation of *d*_1_. Types 1-2 coherent FFLs are shown in (a)-(b), while types 1-2 incoherent FFLs are given in (c)-(d). The red line corresponds to AND gate function, and the green line represents OR gate function. Here we fix *k*_1_ = 0.25.

**Figure 9:**
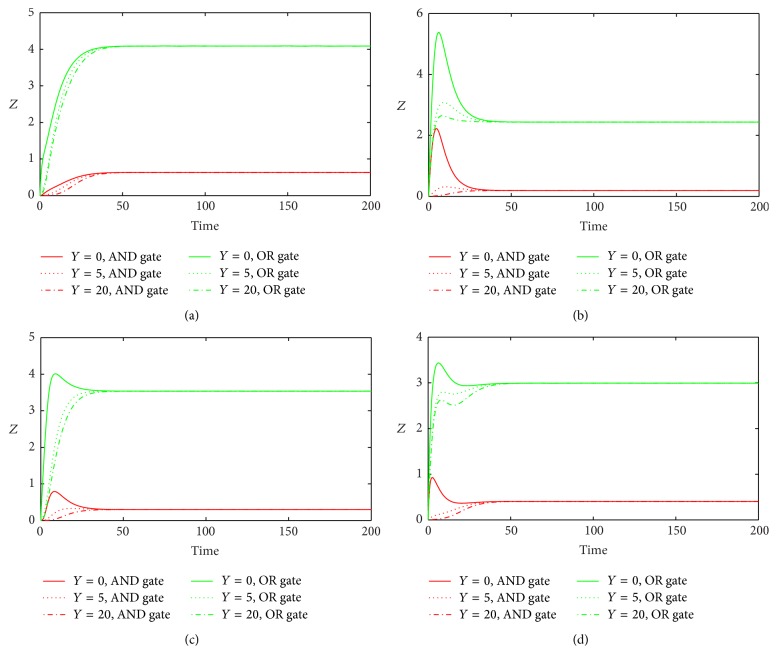
The time evolutions of *Z* in various FFLs with different gate functions in response to the different initial values of miRNA. Types 1-2 coherent FFLs are shown in (a)-(b), while types 1-2 incoherent FFLs are given in (c)-(d). The red line corresponds to AND gate function, and the green line represents OR gate function. Here we choose three different initial values for *Y*, *Y* = 0, *Y* = 5, and *Y* = 20. We fix *k*_1_ = 0.25.

We also investigate the effect of changes in *k*_1_ and *d*_1_ on the dynamical behavior of *Z* (Figures 7 and 8). In Co1 loop, bigger *k*_1_ induces more *Z* with both gate functions, while, in Co2 loop, the situation is opposite; lager *k*_1_ makes less *Z* with both gate functions. This is due to the fact that TF activates target gene directly and promotes it indirectly in Co1 loop, while, in Co2 loop, TF inhibits target gene directly and represses it indirectly. For the cases in In1 and In2 loops with OR gate, both lager *k*_1_ and small *k*_1_ generate nearly the same stationary level of *Z* which is higher than what the original value makes. For the cases in In1 and In2 loops with AND gate, both lager *k*_1_ and small *k*_1_ induce nearly the same stationary level of *Z* which is slightly lower than that induced by the original value. For the variations of *d*_1_ (Figure 8), we get similar results in In1 and In2 loops with both gates, but with the opposite results in Co1 and Co2 loops. Furthermore, we study the effect of different initial values of miRNA on the response of the system (Figure 9). We find that the different initial values of miRNA have no significant influence on the steady state of target gene after it passes the transient state.

## 3. Conclusions

In summary, there are multiple variations of the feedforward loops occurring in the nature based on different types of feedback. Hence, we constructed a mathematical model of FFLs in miRNA-mediated gene transcription network. We introduced four possible structural topologies of FFLs associated with two different gate functions which describe the dynamic interaction between miRNA and TF on gene expression. Dynamical behaviors of model component were investigated by computational simulation. Furthermore, the different features of system's response to simultaneous pulse were investigated. The influence of important parameters on the response of system was also considered. We first identified that only the dynamics of target gene in Co1 loop does not show pulse-like behavior when the synthesis rate of TF is constant. While providing the system with simultaneous pulse, we found that target gene in In2 loop with AND gate is more robust in the presence of stimulus addition, and target gene in In1 loop with AND gate is more stable after an off step of stimulus. Furthermore, we studied the relationship between the response time and maximal activation velocity of miRNA when providing the system with simultaneous pulse. We found that the curve of response time shows nonmonotonic behavior in Co1 loop with OR gate. We further showed that the stationary levels of target gene in all loops were insensitive to the initial value of miRNA.

## Figures and Tables

**Table 1:** The values of parameters in the mathematical model.

Parameter number	Symbol	Value	Description
1	*d*_1_	0.2	Degradation rate of TF
2	*v*_2_	1.0	Maximal activation velocity of miRNA by TF
3	*d*_2_	0.2	Degradation rate of miRNA
4	*v*_3_	1.0	Maximal activation velocity of target gene by TF and miRNA
5	*d*_3_	0.2	Degradation rate of target gene
6	*k*_12_	1.0	Michaelis constant of miRNA by TF
7	*k*_13_	1.0	Michaelis constant of target gene by TF
8	*k*_23_	1.0	Michaelis constant of target gene by miRNA
9	*n*	2	Hill coefficient

**Table 2:** Initial values of the mathematical model.

Parameter number	Symbol	Value	Description
1	*X*	0	Initial value of TF
2	*Y*	0	Initial value of miRNA
3	*Z*	0	Initial value of target gene
